# A first principles investigation on the structural, mechanical, electronic, and catalytic properties of biphenylene

**DOI:** 10.1038/s41598-021-98261-9

**Published:** 2021-09-24

**Authors:** Yi Luo, Chongdan Ren, Yujing Xu, Jin Yu, Sake Wang, Minglei Sun

**Affiliations:** 1 School of Materials Science and Engineering, Southeast University, Nanjing, 211189 Jiangsu China; 2 Department of Physics, Zunyi Normal College, Zunyi, 563002 Guizhou China; 3 College of Science, Jinling Institute of Technology, Nanjing, 211169 Jiangsu China

**Keywords:** Nanoscale materials, Theory and computation

## Abstract

Recently, a new two-dimensional allotrope of carbon (biphenylene) was experimentally synthesized. Using first-principles calculations, we systematically investigated the structural, mechanical, electronic, and HER properties of biphenylene. A large cohesive energy, absence of imaginary phonon frequencies, and an ultrahigh melting point up to 4500 K demonstrate its high stability. Biphenylene exhibits a maximum Young’s modulus of 259.7 N/m, manifesting its robust mechanical performance. Furthermore, biphenylene was found to be metallic with a n-type Dirac cone, and it exhibited improved HER performance over that of graphene. Our findings suggest that biphenylene is a promising material with potential applications in many important fields, such as chemical catalysis.

## Introduction

Carbon exists in different hybridisation states and forms various crystalline materials. Graphene, a two-dimensional (2D) allotrope of carbon, has attracted considerable attention owing to its peculiar properties and promising applications in various fields^1–6^. Since the discovery of graphene, research on graphene analogues has also gained significant interest, and various 2D materials have been theoretically predicted. The predicted 2D carbon materials reportedly exhibit many intriguing properties such as massless Dirac cones^7–9^, semiconducting properties with a sizeable bandgap^10,11^, and even topological properties^12^. In addition to graphene, rare 2D carbon materials, such as graphdiyne^13^, graphtetrayne^14^, naphyne^15^, and phagraphene^16^ have been experimentally synthesised. Recently, a novel carbon allotrope named biphenylene was successfully fabricated^17^; however, its mechanical properties and potential applications are still not completely understood.

In this paper, we report the structural, mechanical, electronic, and catalytic properties of biphenylene obtained by first-principles calculations. This paper is organised as follows: in Sec. 2, we introduce the methods employed; in Sec. 3.1, we discuss the structural features and stability; in Sec. 3.2, the mechanical properties including Young’s moduli, Poisson’s ratio, and fracture strain (strength) are described; in Sec. 3.3, the electronic properties are discussed; in Sec. 3.4, we investigate the catalytic performance of biphenylene for the hydrogen evolution reaction (HER); and in Sec. 4, we summarise the results and draw conclusions.

## Method

First-principles calculations were implemented in the Vienna ab initio simulation package^18^, using the Perdew-Burke-Ernzerhof exchange–correlation functional within the projector-augmented wave method (cut-off energy 800 eV)^19,20^. A Γ-centred 8 × 8 × 1 k-mesh was used to sample the first Brillouin zone. To eliminate the interactions between adjacent layers, a 25 Å thick vacuum space was added perpendicular to the biphenylene network. The energy and ionic force convergence were set to 10^–8^ eV and 10^–4^ eV/Å, respectively. The phonon dispersion calculations were performed using density functional perturbation theory in the Phonopy code^21^. Ab initio molecular dynamics (AIMD) simulations were performed using the canonical ensemble with the temperature regulated by the Nosé−Hoover thermostat^22^. The LOBSTER code is employed to calculate the crystal orbital Hamilton population^23,24^.

## Results and discussion

### Structure and stability

**Figure 1:**
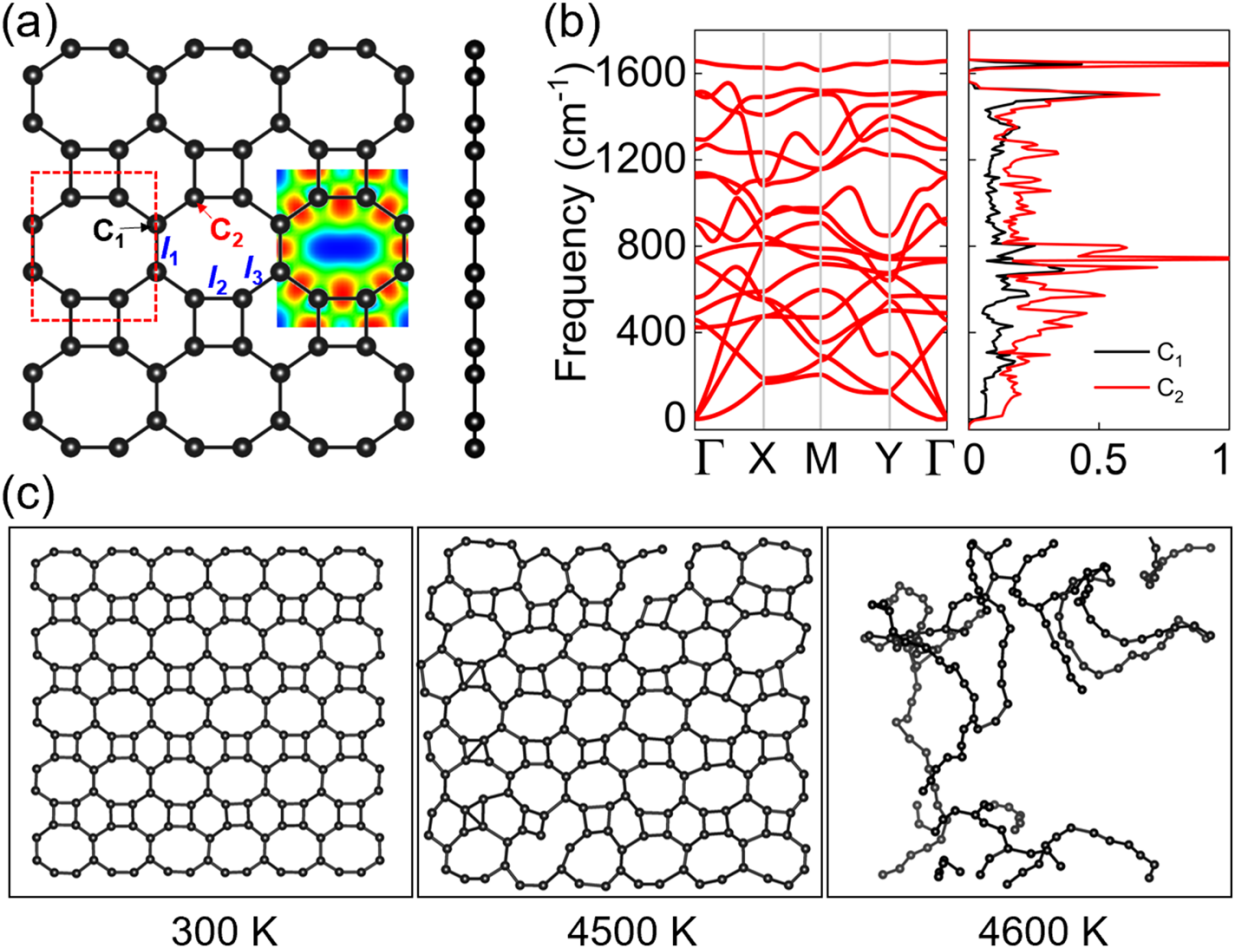
(**a**) Top and side views of the crystal structure of biphenylene; the inset shows the ELF. The primitive cell is marked by red dashed lines; (**b**) phonon dispersion (6 × 5 × 1 supercell); (**c**) results of AIMD simulation at various temperatures.

To establish a reference benchmark, we systematically investigated the structural properties and stabilities of monolayer biphenylene. Figure 1a shows the atomic structure of biphenylene. The unit cell has a rectangular geometry (space group *Pmmm*; group no. 47) with six carbon atoms. The lattice constants of monolayer biphenylene are *a* = 3.76 Å and *b* = 4.52 Å. The *b*/*a* ratio of 1.20 and the presence of different atomic arrangements along the *x* and *y* directions suggest an anisotropic structure. As a non-benzenoid carbon allotrope, biphenylene is constructed from octagonal, tetragonal, and hexagonal rings, which causes slight variations in the carbon–carbon bond lengths. As shown in Fig. 1a, the carbon − carbon bond lengths *l*_1_, *l*_2_, and *l*_3_ are 1.45, 1.46, and 1.41 Å, respectively, which are similar to those of graphene (1.42 Å), indicating the robust structure of biphenylene. The electron localisation function (ELF) from the aspect of the (0 0 1) plane is shown in Fig. 1a to illustrate the bonding behaviour. A value of 1 for the ELF, denoted by red colour, corresponds to perfect localization. We find the highest ELF value in the middle of the carbon−carbon bond is 0.93, indicating that strong covalent bonds exist between the carbon atoms. Noteworthily, the values of integrated crystal orbital Hamilton population at the Fermi level are − 9.35, − 9.03, − 10.07 for the *l*_1_, *l*_2_, *l*_3_, respectively, the smallest value of − 10.07 implies the *l*_3_ bonding is the strongest one.

We demonstrate the stability of biphenylene by means of cohesive energy, phonon spectrum, and AIMD simulations. The cohesive energy *E*_C_ is calculated as (6*E*_carbon_ − *E*_total_)/6, where *E*_carbon_ and *E*_total_ represent the total energies of a carbon atom and a unit cell, respectively. The *E*_C_ value obtained for biphenylene is 7.40 eV/atom, which is higher than that of MoS_2_ (5.02 eV/atom) and boron nitride (7.07 eV/atom) and close to that of graphene (7.85 eV/atom). The phonon dispersion and phonon density of states are shown in Fig. 1b. There are 18 branches (3 acoustic and 15 optical) in the phonon dispersion of biphenylene, and no imaginary frequencies are observed. The maximum frequency is as high as 1657 cm^−1^, indicating good dynamic stability. The phonon states contributed by the C_1_ and C_2_ carbon atoms are coupled in the entire range. The results of AIMD simulations at various temperature are shown in Fig. 1c. No structural distortion, bond breaking, or phase transition is observed at 300 K. Even heat up to 4500 K, the structure is still intact, and finally melt at 4600 K, demonstrating the excellent thermal stability of biphenylene. Overall, our simulation results confirm the excellent stabilities of biphenylene, that is why the biphenylene monolayer can be successfully fabricated experimentally^17^.

### Mechanical properties

The elastic constants were calculated to be C_11_ = 294 N/m, C_22_ = 240 N/m, C_12_ = C_21_ = 91 N/m, and C_66_ = 83 N/m. These values satisfy Born–Huang stability criteria^25^, C_11_C_22_–C_12_^2^ > 0 and C_66_ > 0, suggesting that the biphenylene structure is mechanically stable. We further evaluated the Young’s modulus and Poisson’s ratio of biphenylene. The orientation-dependent Young’s modulus *E(θ)* and Poisson’s ratio *ν(θ)* were determined using the following equations:

1 $$E(\theta ) = \frac{{C_{11} C_{22} - C_{12}^{2} }}{{C_{11} \sin^{4} \theta + \left[ {\left( {C_{11} C_{22} - C_{12}^{2} } \right)/C_{66} - 2C_{12} } \right]\sin^{2} \theta \cos^{2} \theta + C_{22} \cos^{4} \theta }}$$

2 $$\nu (\theta ) = \frac{{C_{12} \sin^{4} \theta - \left[ {C_{11} { + }C_{22} - \left( {C_{11} C_{22} - C_{12}^{2} } \right)/C_{66} } \right]\sin^{2} \theta \cos^{2} \theta + C_{12} \cos^{4} \theta }}{{C_{11} \sin^{4} \theta + \left( {C_{11} C_{22} - C_{12}^{2} } \right)/C_{66} - 2C_{12} \sin^{2} \theta \cos^{2} \theta + C_{22} \cos^{4} \theta }}$$

**Figure 2:**
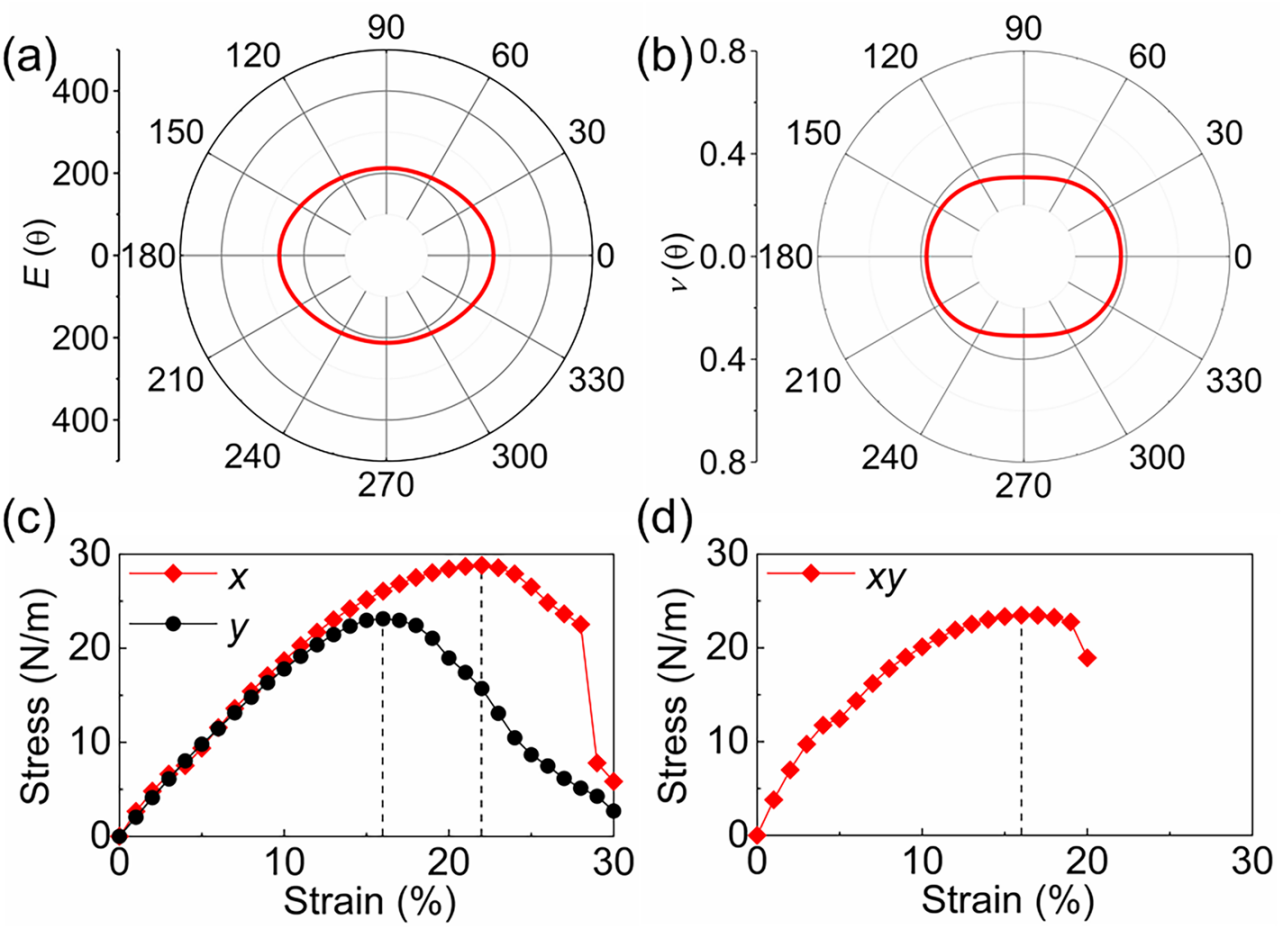
(**a**) Young’s moduli and (**b**) Poisson’s ratio of biphenylene; strain−stress relations along *x* and *y* directions under (**c**) uniaxial and (**d**) biaxial strains.

To elucidate the anisotropic mechanical properties of biphenylene, the polar 2D Young’s moduli and Poisson’s ratio diagrams were calculated. As shown in Fig. 2a,b, the mechanical properties of biphenylene are anisotropic in the plane. For both *E(θ)* and *ν(θ)*, the largest value is along the *x* direction, and the smallest value is along the *y* direction. The Young’s moduli of biphenylene along the *x* (*E*_*x*_) and y directions (*E*_*y*_) are 259.7 and 212.4 N/m (corresponds to 764 and 625 GPa), respectively. The Poisson’s ratio is 0.38 along the *x* direction and 0.31 along the *y* direction. The Young’s modulus of biphenylene is much higher than that of black phosphorene (83 N/m)^26^ and MoS_2_ (123 N/m)^27^, close to that of BN (*E*_*x*_ = *E*_*y*_ = 275 N/m)^28^, and slightly smaller than that of graphene (*E*_*x*_ = *E*_*y*_ = 340 N/m)^29^. These results indicate the robust mechanical properties of biphenylene.

The strain−stress curves under uniaxial (tensile strain from 0 to 30%) and biaxial strains (tensile strain from 0 to 20%) are displayed in Fig. 2c,d, respectively. Figure 2c shows that the fracture strain (strength) is 22% (28.81 N/m) along the *x* direction and 16% (23.13 N/m) along the *y* direction. The phonon dispersions were also employed to confirm the determined fracture strains, and the results are shown in Fig. S1. There are no imaginary frequencies in the phonon spectra until the fracture strains are reduced to 21% along the *x* direction and 14% along the *y* direction under uniaxial strain. Hence, we corrected the fracture strain (strength) to 21% (28.69 N/m) along the *x* direction and 14% (22.34 N/m) along the *y* direction. The fracture strain for biaxial strain (strength) was reduced to 16% (23.50 N/m). After the correction using the phonon spectra (Fig. S1), it was further reduced to 11% (21.07 N/m). Notably, the predicted fracture strengths of biphenylene are larger than those of black phosphorene, which has a fracture strength limit of 10 N/m along the *x* direction and 4 N/m along the *y* direction^30^. Thus, biphenylene exhibits robust mechanical properties.

### Electronic properties

**Figure 3:**
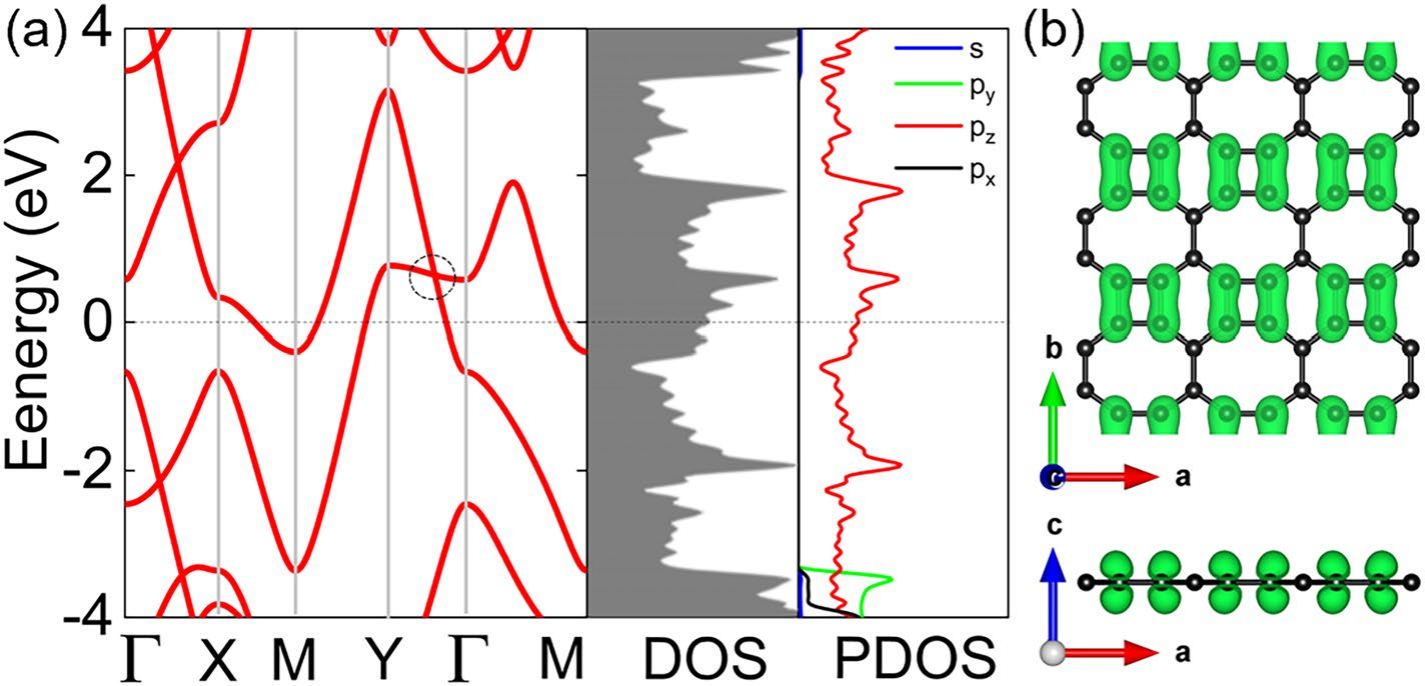
(**a**) Band structure (left panel), density of states (middle), and projected density of states (right panel) of biphenylene; (**b**) the partial charge density of biphenylene, the value of the isosurface is set to 0.01 e Å^-3^.

The band structure in Fig. 3 shows several bands across the Fermi level, indicating that biphenylene is metallic; this agrees well with the experimental d*I*/d*V* spectra reported previously^17^. The total density of states shown in Fig. 3 indicates several peaks across the Fermi level, for example, at 0.22 and 0.58 eV. We further analysed the projected density of states and found that the states near the Fermi level are mainly contributed by the p_*z*_ orbitals of the carbon atoms. As the sp^2^-hybridized allotrope of carbon, the formed n-type Dirac cone approaches (0.64 eV above) the Fermi energy level along the Y − Γ line. Such tilted Dirac cones have been reported in other 2D materials such as Be_5_C_2_^31^, *Pmmn* boron^32^, and FeB_3_^33^. The origin of the Dirac cone can be attributed to the out-of-plane p_*z*_ orbitals, as shown in Fig. 3b.

### Catalysis of HER

**Figure 4:**
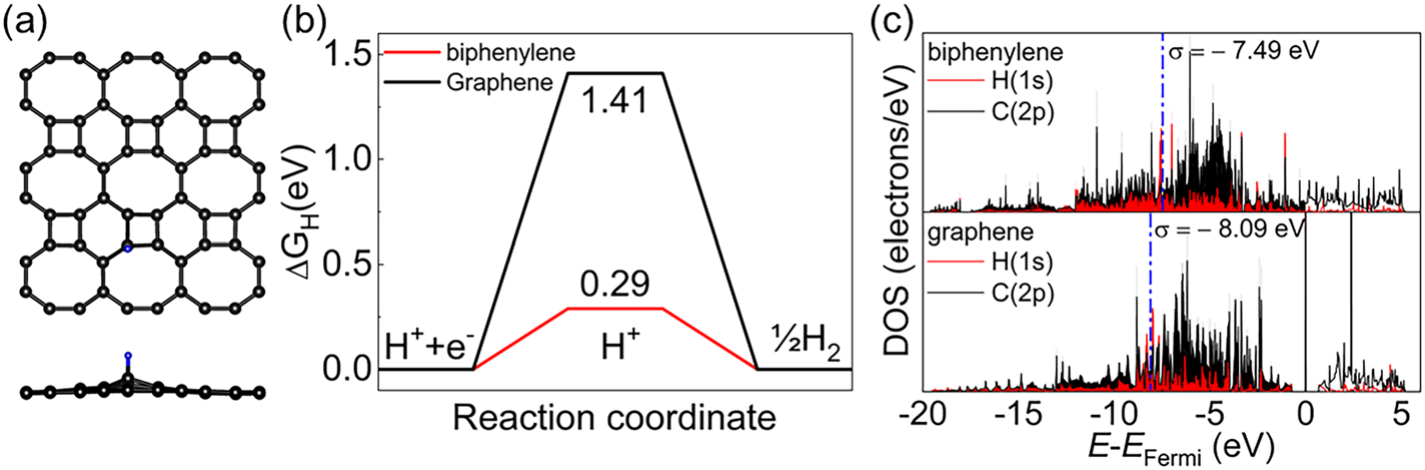
(**a**) Top and side views of the most stable adsorption configuration; (**b**) Gibbs free energy for HER; (**c**) projected density of states for H atom adsorption on biphenylene and pristine graphene.

As biphenylene shows good metallic properties, it can be potentially used as a catalyst. Hence, we probed the catalytic performance of biphenylene by the HER. The Gibbs free energy change (Δ*G*_H_ = Δ*E* + Δ*E*_zpe_ + TΔ*S*, standard conditions) of the intermediate (H^*^) in the following two reactions is considered for evaluating the HER performance of the catalyst.

5 $$* \, + {\text{ H}}^{ + } + {\text{ e}}^{ - } \to {\text{H}}^{*} ,$$

6 $${\text{H}}^{*} + {\text{ H}}^{ + } + {\text{ e}}^{ - } \to {\text{H}}_{{2}} + \, *,$$

where Δ*E* is the adsorption energy of H^*^ species, Δ*E*_zpe_ is the change in the zero-point energies, *T* is 298.15 K, Δ*S* is the difference in the entropy before and after adsorption, and * is the active site. We considered all possible adsorption sites for a single H atom on a 3 × 3 biphenylene supercell. Figure 4a shows the most stable adsorption configuration. According to Fig. 4b, the Gibbs free energy for HER is 0.29 eV at U = 0 eV, which is much smaller than that of 2H-MoS_2_ (2 eV)^34^, better than that of g-C_3_N_4_ (0.54 eV)^35^ and WSSe (0.58 eV)^36^, and comparable to that of recently reported Pd_4_S_3_Te_3_ (0.18 eV)^37^. The Gibbs free energy change of pristine graphene for HER is also shown in Fig. 4b. Notably, biphenylene exhibits a significantly higher catalytic activity than pristine graphene (ΔG_H_ = 1.41 eV). To further reveal the improved catalytic performance of biphenylene, we introduced the σ centre theory ($\varepsilon_{\text{s}} = \frac{{\int_{ - \infty }^{\infty } {n_{s} \left( \varepsilon \right)\varepsilon d\varepsilon } }}{{\int_{ - \infty }^{\infty } {n_{s} \left( \varepsilon \right)d\varepsilon } }}$). According to the results shown in Fig. 4c, biphenylene has a higher σ centre position (− 7.49 eV) than graphene (− 8.09 eV), which indicates stronger hydrogen bonding ability (a strong correlation between σ centre and Δ*G*_H_ values is shown in Fig. S2). These results indicate that biphenylene has significant potential for catalysing HER.

## Conclusion

Based on first-principles calculations, we systematically explored the structural, mechanical, electronic, and HER properties of biphenylene. Our results show that monolayer biphenylene is stable with a large cohesive energy (7.40 eV/atom) and is characterised by a phonon spectrum with no imaginary frequencies and an ultrahigh melting point up to 4500 K. In addition, a maximum Young’s modulus of 259.7 N/m reveals its robust mechanical properties. The determined fracture strains (strengths) are 21% (28.69 N/m) and 14% (22.34 N/m) along the *x* and *y* directions under uniaxial strain, respectively. Under biaxial strain, the fracture strain (strength) is reduced to 11% (21.07 N/m). The metallic nature with a n-type Dirac cone of biphenylene along with its outstanding performance in HER (Δ*G*_H_ = 0.29 eV) demonstrate the potential of biphenylene as a catalyst.

## Supplementary Information

Supplementary Information.
