# Supplementary material for: A first principles investigation on the structural, mechanical, electronic, and catalytic properties of biphenylene
Source: Sci Rep. 2021 Sep 24;11:19008. doi: 10.1038/s41598-021-98261-9 (PMC8463688; doi:10.1038/s41598-021-98261-9)
Supplement: Supplementary file 1 — Supplementary Information. [file 41598_2021_98261_MOESM1_ESM.docx]

**Supporting information for**

**A first principles investigation on the structural, mechanical, electronic, and catalytic properties of biphenylene**

*Yi Luo,^a^ Chongdan Ren,^b^* *Yujing Xu,^a^ Jin Yu,^a*^ Sake Wang,^c*^and Minglei Sun^a*^*

*^a^School of Materials Science and Engineering, Southeast University, Nanjing, Jiangsu 211189, China*

*^b^Department of Physics, Zunyi Normal College, Zunyi, Guizhou, 563002, China*

*^c^College of Science, Jinling Institute of Technology, Nanjing, Jiangsu, 211169, China*

*Corresponding author: Jin Yu (Email*[*: yujin@seu.edu.cn)*](mailto::%20yujin@seu.edu.cn))*, Sake Wang (Email:* [*IsaacWang@jit.edu.cn)*](mailto:IsaacWang@jit.edu.cn))*, Minglei Sun (Email: mingleisun@outlook.com)*


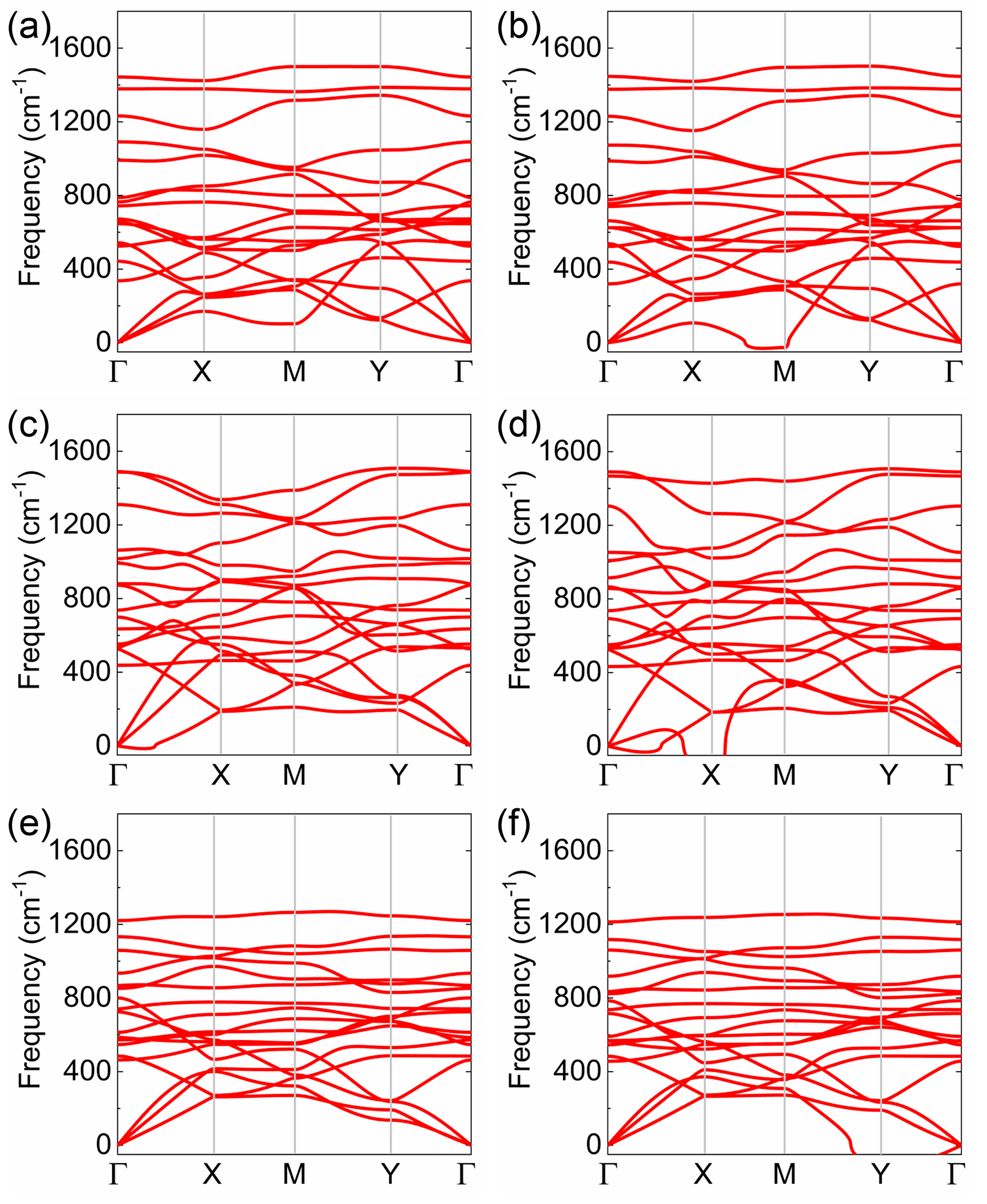


Figure S1. The phonon spectra for biphenylene under (a) 21%, (b) 22% uniaxial strain along the *x*-direction, under (c) 14%, (d) 15% uniaxial strain along the *y*-direction; and under (e) 11%, (f) 12% biaxial strain.


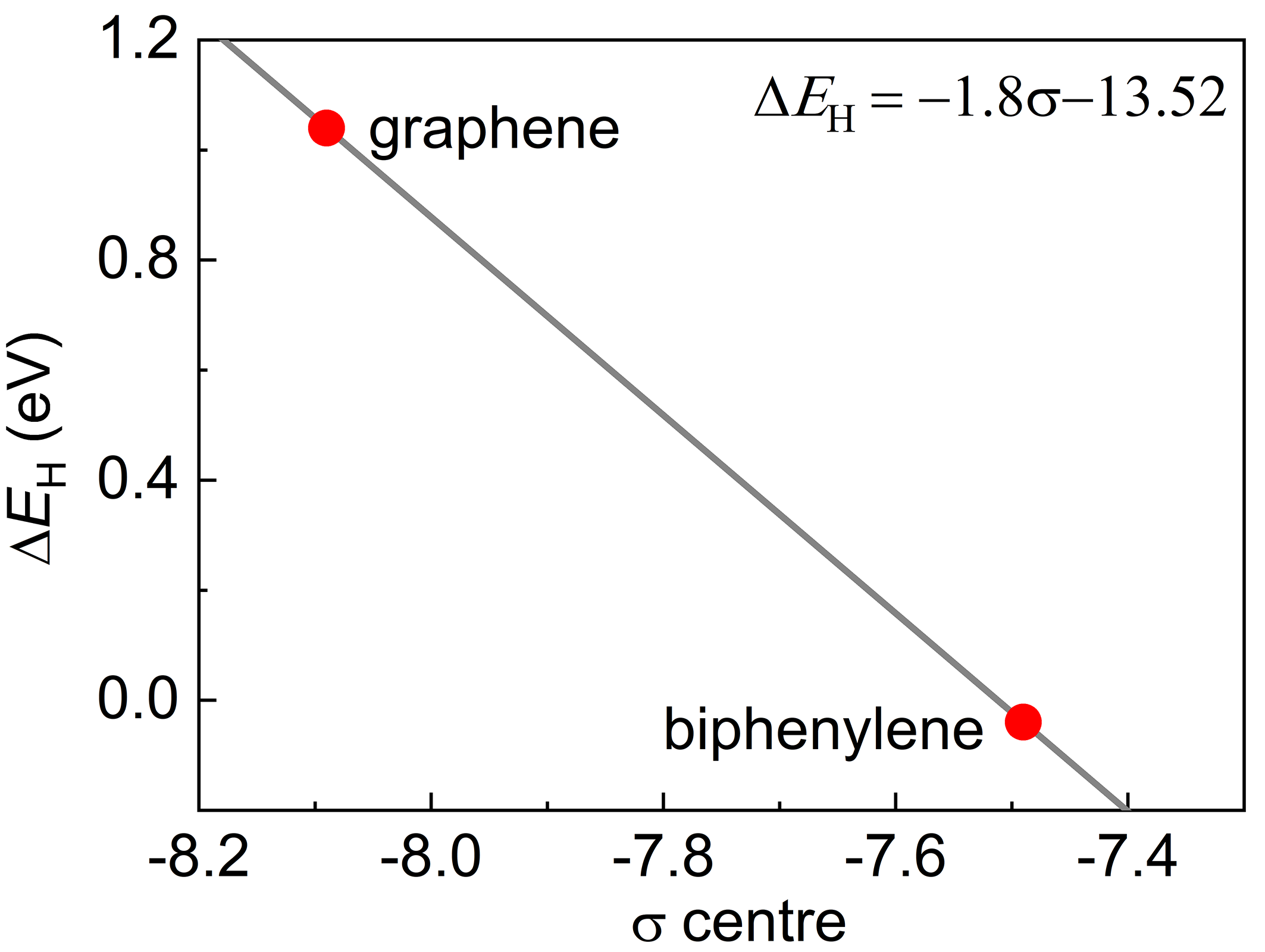


Figure S2. Relationship between Δ*E*_H_ and σ band center of biphenylene and graphene. The Δ*E*_H_ is the bonding energies of H atom on biphenylene and graphene.
